# Beyond lymphopenia, unraveling radiation-induced leucocyte subpopulation kinetics and mechanisms through modeling approaches

**DOI:** 10.1186/s13046-023-02621-4

**Published:** 2023-02-22

**Authors:** Thao-Nguyen Pham, Julie Coupey, Serge M. Candeias, Viktoriia Ivanova, Samuel Valable, Juliette Thariat

**Affiliations:** 1 Normandie Univ, UNICAEN, CNRS, ISTCT, GIP CYCERON, 14000 Caen, France; 2 Laboratoire de Physique Corpusculaire UMR6534 IN2P3/ENSICAEN, Normandie Université, Caen, France; 3 Univ. Grenoble Alpes, CEA, CNRS, IRIG-LCBM-UMR5249, 38054 Grenoble, France; 4 Department of Radiation Oncology, Centre François Baclesse, Caen, Normandy France

**Keywords:** Radiotherapy, Modeling, Leucopenia, Lymphopenia, Myelosuppression

## Abstract

Leucocyte subpopulations in both lymphoid and myeloid lineages have a significant impact on antitumor immune response. While radiation-induced lymphopenia is being studied extensively, radiation effects on lymphoid and myeloid subtypes have been relatively less addressed. Interactions between leucocyte subpopulations, their specific radiation sensitivity and the specific kinetics of each subpopulation can be modeled based on both experimental data and knowledge of physiological leucocyte depletion, production, proliferation, maturation and homeostasis. Modeling approaches of the leucocyte kinetics that may be used to unravel mechanisms underlying radiation induced-leucopenia and prediction of changes in cell counts and compositions after irradiation are presented in this review. The approaches described open up new possibilities for determining the influence of irradiation parameters both on a single-time point of acute effects and the subsequent recovery of leukocyte subpopulations. Utilization of these approaches to model kinetic data in post-radiotherapy states may be a useful tool for further development of new treatment strategies or for the combination of radiotherapy and immunotherapy.

**Supplementary Information:**

The online version contains supplementary material available at 10.1186/s13046-023-02621-4.

## Background

Leucocyte subpopulations in both lymphoid and myeloid lineages exert an essential impact on tumor regulation. Leucocytes are part of the immune system, which protects the body against foreign invaders, as well as cancer [1]. Homeostasis is the mechanism that helps maintain stable levels of leucocytes in the blood against any variability [2]. Homeostasis is crucial to a proper functioning of the immune system. Prediction of leucocyte kinetics and homeostasis in cancer patients is paramount, given that the immune system plays an essential role in identifying malignant cell properly and reacting against them, which underlies the use of immunotherapies in cancer treatment [3]. Leucocytes are divided into lymphoid or myeloid cells, originating from their common hematopoietic stem cells within the bone marrow [4]. Both leucocyte lineages participate to cancer surveillance. Lymphopenia is associated with poor prognosis, while favoring tumor development [5, 6]. Myeloid cells either promote or control tumor growth, depending on their subtype [7, 8]. Neutrophils are the most abundant myeloid cells, which have similarly emerged as cancer regulators. Indeed, a high circulating neutrophil to lymphocyte ratio likely is a robust prognostic factor of poor clinical outcome in various cancers [9].

Radiotherapy, which is used in over 50% of cancer patients, exerts inevitable, yet usually manageable, deleterious effects on normal healthy cells along the radiation beam path. Until recently, the suppressive effect of radiotherapy on the immune system has been largely ignored in routine practice. Radiation-induced immune suppression was investigated in the 1970’s, at the time of the premises of immunotherapy, then forgotten for about 30 years until a recent rebound in interest in new immunotherapy modalities [10, 11]. The synergy between radiotherapy and immunotherapy is currently undergoing intense investigations. Controlling radiation-induced leucopenia is likely critical for optimizing the synergistic effect of these two treatments [3]. However, the radiation-induced leucopenia has scarcely been included in models estimating the probability of undesirable effects on normal tissues represented by normal tissue complication probability (NTCP) [12]. Among leucocytes, lymphopenia is by far the most popular issue due to its frequency, duration, and depth of lymphocyte counts reduction. Lymphoid cells appear more radiosensitive than myeloid cells, and radiation-induced lymphopenia occurs more often than myelosuppression [13]. However, myeloid cells contribute to prognosis and radiation response in tumors, whereas their kinetics in the blood have been barely investigated [14]. Radiation effects on leucocytes are indeed heterogeneous, and both of the lymphoid and myeloid lineages can impact prognosis and response to immunotherapy [15, 16].

Mathematical modeling has become increasingly useful in medical research as a tool to understand disease mechanisms, suggest optimal treatment modalities, and predict treatment outcomes [17]. Recent publications have highlighted the emerging role of mathematical modeling in optimizing the synergistic action of combined radiotherapy and immunotherapy in tumor control. Mathematical modeling of longitudinal data of leucocyte subpopulation kinetics following irradiation represents a potential approach enabling to predict radiotherapy effects, as well as to further optimize combinations of radiotherapy-immunotherapy, based on understanding physiological leucocyte homeostasis. The number and relative proportion of leucocyte subpopulations vary widely across cancer patients, depending on inflammation or cancer progression. Therefore, detailed leucocyte subpopulation analysis may better inform about prognosis and mechanism of treatment responses in comparison with global approaches involving limited time points [1]. A good understanding of the known biological process is critical for developing mathematical modeling, with its interpretation and application.

This review sought to evaluate the mechanisms and kinetics of radiation-induced leucopenia, additionally addressing the relationships between lymphoid and myeloid lineages, as well as the dynamics between tissues and blood based on mathematical modeling in order to optimize radiotherapy and radiotherapy-immunotherapy combinations.

Appropriate references were identified on the basis of: 1) medicine, biology, and modeling text books for general definition of leucocytes and their subpopulations, their roles in immune response, and for definition of different modeling strategies; 2) searches of PubMed using the search terms “Leucocyte homeostasis”, “Radiation-induced lymphopenia”, “Radiation-induced myelosuppression”, “Radiosensitivity”, “Mathematical modeling”, and “Biological modeling” from 1970 until 2021. Only papers published in English were reviewed. The final reference list was generated on the basis of originality and relevance to the broad scope of this work.

## Main text

### The lymphoid population

Mature lymphoid cells, i.e., lymphocytes, are subdivided into T-cells, B-cells, and NK-cells based on protein-complexes on their surface. In humans, T-cells (expressing T-cell receptors [TCR] comprise 40–60% of the total circulating pool of lymphocytes, and B-cells (expressing B-cell receptors BCR) 20–30% [1, 18] of the pool, while NK-cell proportions range from 4 to 28%. The T-cell population is subdivided into CD4 + (including helper and regulatory T-cells) and CD8 + T-cells (cytotoxic T-cells) [1]. Reduced lymphocyte counts in blood, i.e., lymphopenia, has been correlated with poor survival in patients with solid tumors [10].

#### Lymphoid lineage function

T-cells and B-cells participate in adaptive immune responses, and different lymphocyte types play specific roles: T-cells synthesize and release cytokines or kill their target cells. B-cells mediate immune responses by releasing antibodies [1]. NK-cells belong to the innate immune system, and they mediate anti-viral and anti-tumor responses [19].

Considering tumor responses, T-cells can identify and eradicate tumors through TCR recognition of tumor-associated antigens [20, 21]. B-cells participate in suppressing tumor progression by secreting immunoglobulins, promoting T-cells responses, and activating NK-cells [22]. NK-cells are able to kill tumor cells. NK-cells and helper T-cells promote cytotoxic T-cells to differentiate into effective cytotoxic T-cells that eliminate tumor cells. These anti-tumor effects are suppressed by regulatory T-cells [20].

#### Physiology of lymphoid populations

##### Production, maturation, and distribution

Lymphocyte progenitors are produced in the bone marrow (Fig. 5A) [23]. B-cell maturation is initiated in the BM and finalized in the spleen, while T-cells are generated in the thymus after migration of lymphocyte progenitors. Naïve (non-activated mature) lymphocytes either circulate in the blood or home into secondary lymphatic organs (SLOs), such as the spleen and lymph nodes (LNs). Naïve T-lymphocytes become activated lymphocytes by interaction with antigen presenting cells in LNs. Activated lymphocytes move to inflammatory sites so as to participate in immune responses. Thereafter, they either die by apoptosis or recirculate [24, 25]. The modeling of lymphocyte recirculation based on available data in rodents has generated quantitative estimates of migration rates and residence time of lymphocytes in major organs. Indeed, mean transit time was shown to be long in SLOs, exceeding 2 h in the spleen, and 10 h in LNs, compared with less than a minute in the lung and liver [26].

Only a small number of lymphocytes is indeed present in circulating blood at any time. In rodents, 5% of lymphocytes can be found in the blood, 25% in the spleen, and 70% in the LNs or other lymphoid organs [27]. Simulated instantaneous lymphocyte distribution in rodents is shown in Table 1. In humans, no direct method allows to estimate the lymphocyte distribution in organs. Extrapolation of rodent data to humans based on organ size of both species has revealed that about 2% of lymphocytes reside in the blood at any time (Table 1) [28].

**Fig. 1:**
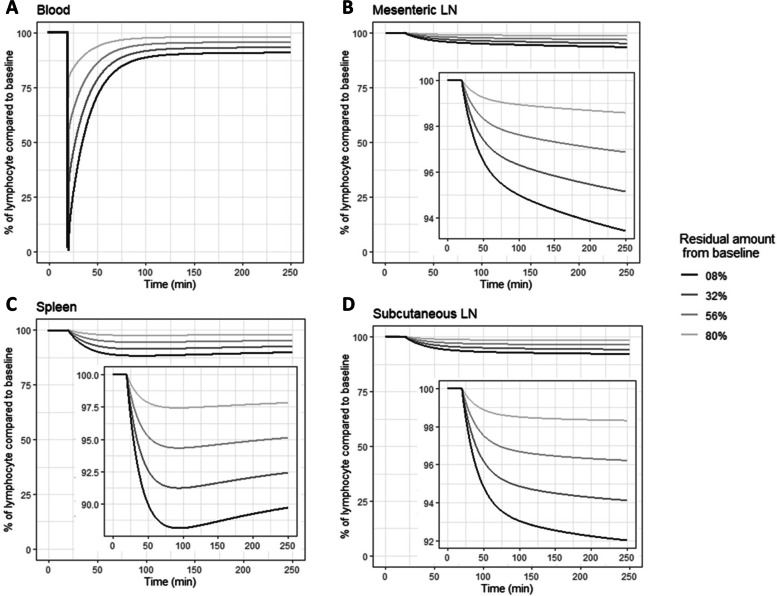
Simulation of lymphocyte kinetics in the blood and SLO after a sudden drop in blood, using lymphocyte recirculation model developed by Ganusov and Auerbach, 2014 [26]. Legend: Following a sudden drop in the blood compartment at 25 min, lymphocytes in blood recovered to higher than 80% of their initial level in less than 200 min. This recovery occurred because of the recruitment of lymphocytes homing in SLOs. According to this simulation, the lymphocytes in SLOs (spleen and LNs) decrease gradually following their reduction in blood. Details of simulation are provided in the supplementary materials (Supplementary Fig. 3)

Simulation from recirculation models likewise showed that following a sudden drop, the blood lymphocyte counts nearly reached their initial levels in less than 3 h via lymphocyte recruitment from lymphatic organs (Fig. 1).

**Table 1:** Lymphocyte distribution in rodents (simulation explained in supplementary data: Supplementary Fig. 1 and Supplementary Fig. 2) and human [28]

Organs	Blood	Spleen	Other secondary lymphatic organs	Others
% of lymphocytes (Rodents)	4.15	33.21	58.08	4.56
% of lymphocytes (Human)	2.2	15.2	45.6	37.0

##### Homeostasis

The mechanisms of lymphocyte homeostasis were mostly studied using experiments in rodents, prior to these data being extrapolated to humans. Homeostasis plays an essential role in lymphocyte recovery after acute lymphopenia. The control of both T-cells and B-cells is independent [29].

**Fig. 2:**
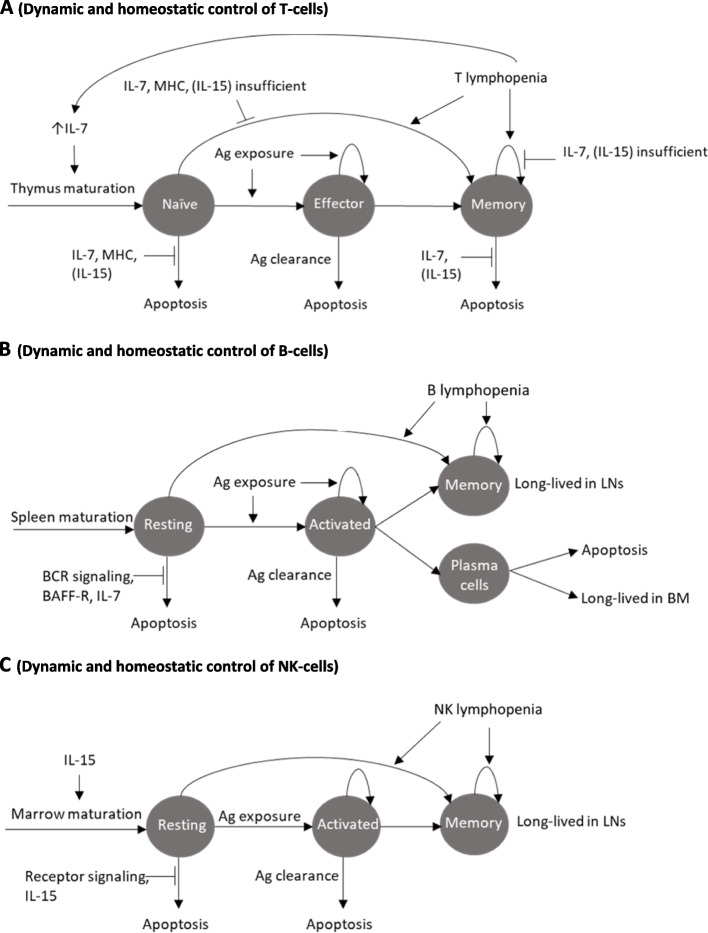
Dynamic and homeostatic control of lymphocytes in the periphery. Legend: Ag, LN, IL, and BAFF-R stand for antigen, lymph node, interleukin, and B-cell activating factor receptor, respectively. **A** Dynamic and homeostatic control of T-cells in the periphery. In rodents, the naïve T-cell level is maintained based on input to the thymus and survival signals, which are controlled by IL-7 and TCR signaling with MHC. The memory T-cell level is maintained by differentiation input from the naïve cell population and survival signals controlled by IL-7. Memory cells can proliferate or differentiate from the naïve population in case of lymphopenia. Effector cells differentiate from naïve T-cells following immune stimulation. Most effector cells die shortly after stimulation by apoptosis, and only a few differentiate into memory cells. **B** Dynamic and homeostatic control of B-cells in the periphery. The resting B-cell level is maintained by input from the spleen and survival signals, controlled by BCR signaling and B-cell activating factor receptor (BAFF-R). Activated B-cells either die after immune stimulation or differentiate into long-lived populations. The long-lived memory population can expand by proliferation or differentiation from the naïve population in case of lymphopenia. **C** Dynamic and homeostatic control of NK-cells in the periphery. The resting NK-cell level is maintained by input from primary lymphatic organs and survival signals, which are controlled by receptor signaling and IL-15. Activated NK-cells either die after immune stimulation or differentiate into long-lived populations

Mature T-cells comprise naïve and memory T-cells. Naïve T-cells are small cells, which mature in the thymus before entering peripheral blood circulation. Once activated, these T-cells travel to extra-lymphoid effector sites where they exert an adaptive immune function. Naïve and memory T-cell counts are governed by independent homeostatic mechanisms to preserve the response to any novel infection via naïve T-cells, whilst ensuring efficient memory responses against known antigens. Studies in rodents suggested that thymus production actually exceeds the quantitative requirements to replenish T-cell counts in the peripheral pools [30]. Naïve T-cell homeostasis is mainly governed by cell survival, which is controlled by cytokine interleukin (IL)-7, and TCR binding to major histocompatibility complex (MHC) molecules. For activated T-cells, survival is mainly controlled by IL-7,yet independent from MHC. T-cell homeostasis also depends on sub-populations’ integrity and presence of regulatory T-cells. The mature T-cell niche depends on outputs from the thymus in rodents or on homeostatic proliferation in humans [31]. Lymphopenia triggers lymphopenia-induced proliferation in order to replenish the depleted T-cell niches [32]. However, lymphopenia-induced proliferation actually expands the memory population, whereas it is insufficient to replenish the naïve T-cell population. T-cells homeostasis has been summarized in Fig. 2A.

The homeostasis regulation of CD4 + and CD8 + populations does not occur independently. Studies in rodents showed that loss of either CD4 + or CD8 + T-cell subsets is likely compensated one by another so that the overall T-cell population size is preserved [33].

Mature B-cells comprise resting and activated B-cells. Resting B-cells are activated through antigen binding with BCR. Antigenic peptides can then be loaded onto MHC complexes; these antigen/MHC complexes are recognized by activated helper T-cells, thereby triggering adaptive immune responses. Activated B-cells can differentiate into plasma cells (either short-lived and dying after infection, or long-lived in the bone marrow for antibody production) or memory B-cells (long-lived in LNs for antibody production) [34]. The resting and activated B-cell populations undergo independent homeostatic regulation. The B-cell production within the bone marrow exceeds the requirement for B-cell counts in the periphery in rodents. Yet, B-cell homeostasis is maintained mainly by their survival in the periphery rather than by bone marrow production [35]. BCR signaling is vital for B-cell survival. B-cells also undergo lymphopenia-induced proliferation following lymphopenia resulting in enlargement of the long-lived population. B-cell homeostasis has been summarized in Fig. 2B.

Mature NK-cell homeostasis is dependent on IL-15. Following immune stimulation by infections, NK-cells are activated, undergo proliferative expansion, and become long-lived memory cells. NK-cells homeostasis has been summarized in Fig. 2C.

#### Impact of irradiation: radiobiological considerations

Lymphocytes are radiosensitive cells in mammals. Cellular radiosensitivity is measured either directly by cell death biomarkers (annexin V/propidium iodide assay for apoptosis/necrosis), using cell surviving fraction, or indirectly by chromosomal aberrations.

In humans, lymphocyte surviving fraction following a single dose of 2 Gy is about 90% in vivo [36]. The survival of lymphocytes is heterogenous across different lymphocyte types (T-cells, B-cells, or NK-cells). In both humans and rodents, B-cells are the most radiosensitive lymphocytes [37, 38]. Among T-cells, CD4 + T-cells were shown to be more sensitive than CD8 + T-cells [39]. NK-cells are more resistant to radiation in comparison with other lymphocyte types, with similar surviving rates of NK-cells at 2 Gy in irradiated and non-irradiated cells [40].

**Fig. 3:**
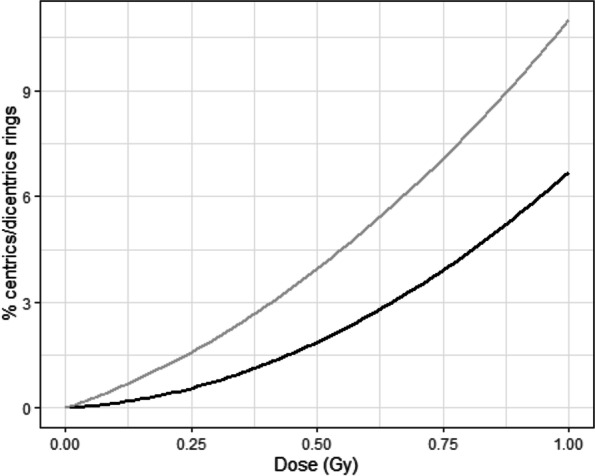
Quadratic linear regression curve describing occurrence of chromosome aberrations in human lymphocytes (grey curve) and neutrophils (black curve) following in vitro irradiation [41, 42]. It shows that chromosome aberrations occur at a higher rate in lymphocytes compared with neutrophils

Radiation induces chromosome damage and aberration like centric/dicentric rings in lymphocytes in the metaphase. Aberrant chromosomes divide unevenly among daughter cells, which then undergo delayed “mitotic death” after a few generations following radiation exposure. Chromosome aberrations are dose-dependent (Fig. 3) [41, 42].

Irradiation may be either restricted to the tumor site or involve sites of probabilistic tumor involvement. A distinct situation is total body irradiation conditioning before bone marrow transplantation (usually in leukemias). In all Rhesus macaques receiving a single dose of 6 Gy, severe lymphopenia occurred immediately. In animals surviving 15 days following irradiation, absolute lymphocyte counts represented 3% of pretherapeutic treatment levels. Recovery occurred slowly to reach stable levels within 100–300 days, following a transient rebound effect [43, 44]. Homeostasis after a single radiation dose takes up to a year to fully recover. In rodent models, lymphocytes isolated from irradiated animals showed a reduction in proliferation and cytokine secretion through reduction of TCR activation [45].

Radiation-induced lymphopenia arises regardless of radiotherapy modality and tumor site. The incidence of Grade 3–4 radiation-induced lymphopenia (total lymphocyte counts < 500 for normal values of 1000—4800 cells/µL) was reported in 89% of cervix cancer patients, and 20–40% of brain tumor patients. Radiation-induced lymphopenia has been associated with poor prognosis in many tumors [10]. Fractionated irradiation causes more severe radiation-induced lymphopenia in comparison with single dose treatment due to lymphocyte redistribution from SLOs into the blood during fraction intervals. Cranial irradiation in leukemic children was connected with a more severe radiation-induced lymphopenia with increasing fraction number for a same total radiation dose. Until now, the precise mechanisms of radiation-induced lymphopenia are still unclear. One of the hypotheses is that radiation-induced lymphopenia is accounted for by the exposure of blood circulating into radiation beams during radiotherapy. Simulations using a model of glioblastoma demonstrated a higher impact in case of fractionated irradiation [46].

### The myeloid population

The myeloid lineage consists of granulocytes (neutrophils, basophils, and eosinophils), monocytes (macrophages), megakaryocytes (platelets), and dendritic cells [4, 47]. The most abundant granulocytes in mammals are the neutrophils [1, 48]. The myeloid population also comprises myeloid-derived suppressor cells, which are either immature monocytes or neutrophils. Myeloid-derived suppressor cells only appear in the blood in persistent myelopoiesis induced by pathological conditions including cancer, which will not be further discussed here[49, 50].

#### Myeloid lineage function

Myeloid cells participate in the innate immune responses. Neutrophils provide an efficient defense barrier against pathogens [1]. Monocytes are phagocytic cells, which home into tissues in order to differentiate into macrophages following infection and tissue damage or macrophage depletion [51].

Myeloid cells play an essential role in tumor response. Neutrophils, depending on their phenotype N1 or N2, exert either tumor-promoting or anti-tumor effects [52]. N1 neutrophils promote tumor angiogenesis. N2 neutrophils participate in tumor elimination by antibody-dependent mechanisms, activation of antitumor adaptive immune mechanisms, as well as cytokine secretion [50, 53]. Monocytes differentiated into M1-macrophages or M2-macrophages in tissues likely exert anti-tumor effect or promote tumor growth, respectively [54, 55].

#### Physiology of myeloid populations

##### Production, maturation, and distribution

Myeloid cells are produced via myelopoiesis, and they differentiate into different cell lines within the bone marrow. Myelopoiesis is stimulated in response to cytokine signals and infectious stimuli. In healthy individuals, myelopoiesis stimulation comes to an end when the stimuli vanish. Persistent myelopoiesis can be observed in cases of chronic infection, inflammation, or cancer.

Neutrophils develop within the bone marrow from the hematopoietic stem cells (HSC) into three steps: proliferation, maturation, and function acquisition (about 14, 6.5, and 2.5 days, respectively). After maturation, neutrophils participate in innate immune responses [56]. Mature neutrophils in their terminal differentiation state recover after neutropenia in a proliferation-dependent way before being released from the bone marrow in the blood. Neutrophil half-life is relatively short of about 3–12 h [57]. Following inflammation, neutrophils are rapidly recruited to injury sites so as to participate in innate immune responses.

After maturation, monocytes stay within the bone marrow for about 1.6 days before being released into the blood. Classical monocytes represent 99% of monocytes, which either migrate into tissues and differentiate into macrophages and then contribute to immune responses, or they die by apoptosis within a day. Non-classical monocytes called immediate monocytes, or also long-lived monocytes, stay within the blood for 4.3 and 7.4 days, respectively [58]. In rodents, 40% of monocytes are distributed in the blood, and 60% are dispersed in marginating pools [59]. In humans, monocytes are in the blood and the spleen, but their largest reservoir under homeostatic conditions is the bone marrow [60].

##### Homeostasis

**Fig. 4:**
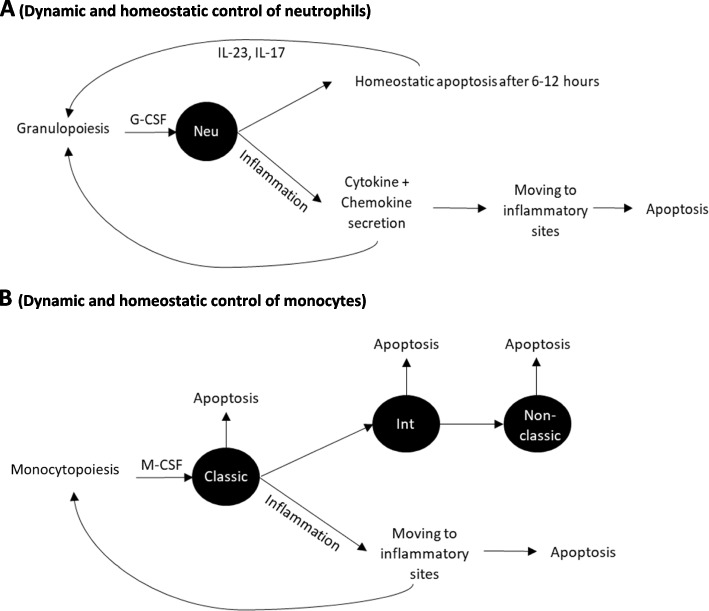
**A** Dynamic and homeostatic control of neutrophils. Legend: Neu: neutrophil. Without any immune stimulation, the neutrophil level in the blood is controlled by a balance between input from bone marrow and cell survival, which is regulated by cytokines, such as G-CSF, IL-23, and IL-17. The homeostasis is disrupted by immune stimulation, as observed in inflammation. **B** Dynamic and homeostatic control of monocytes. Legend: Classic, Int, and non-classic: classical, intermediate, and non-classical monocytes, respectively. Without immune stimulation, monocyte levels in the blood result from a balance among inputs from the bone marrow, cell survival, and differentiation into long-lived monocytes. The homeostasis is disrupted by immune stimulation, as observed in inflammation

Myeloid cell homeostasis is regulated by multiple factors, including differentiation and proliferation of precursor cells within the bone marrow, release into the blood, margination in organs, as well as apoptosis [61]. For neutrophils, multiple cytokines, including granulocyte colony-stimulating factor (G-CSF), macrophage colony-stimulating factor (M-CSF), granulocyte–macrophage colony-stimulating factor (GM-CSF), IL-6, IL-3, and IL-17, promote progenitor proliferation and differentiation (Fig. 4A). G-CSF receptor deficiency leads to profound neutropenia. Neutrophils release from the bone marrow into the blood is inhibited by interactions of stromal derived factor-1 (SDF1) with the chemokine receptor CXC-receptor 4 (CXCR4). CXCR4 down-regulation by G-CSF is associated with neutropenia, thereby increasing mature neutrophil residency in bone marrow [62]. Phagocytosis of apoptotic neutrophils regulates granulopoiesis via IL-23 and IL-17 [63].

The production of monocytes in the bone marrow is governed by growth-stimulating cytokines, such as IL-3, SCF, GM-CSF, and M-CSF [64]. Monocyte release from the bone marrow into the blood depends on C–C chemokine receptor 2 (CCR2). CCR2 deficiency results in monocytopenia (Fig. 4B).

#### Impact of irradiation: radiobiological considerations

Myeloid cells are less radiosensitive than lymphoid cells. Murine monocytes and myeloid progenitor cell lines display almost no decline 24 h after 4 Gy in vitro irradiation [13]. In humans, in vitro studies have revealed significant numbers of chromosome aberrations in neutrophils following 0.5 Gy single-dose irradiation. The proportion of dicentric rings was shown to increase with the radiation dose following a linear quadratic function, yet to a lesser extent in neutrophils than lymphocytes (Fig. 3) [41, 42].

Marked reduction in myeloid cells has been observed after total body irradiation. In tumor-free rhesus macaques, a 6 Gy single dose of total body irradiation resulted in a neutrophil nadir of 10% and recovery within a month. Radiation-induced neutropenia occurs after extended field radiotherapy involving a large portion of the bone marrow or with concomitant use of myelosuppressive chemotherapy agents [15]. High neutrophil blood counts have been linked to more aggressive cancer and detrimental outcomes in several solid tumors. Predicting neutropenia following radiotherapy as a prognostic factor could be instrumental in optimizing treatments [52, 53].

### Balance between leucocyte subpopulations

Balance between myeloid and lymphoid populations is critical for immune activities. Skewing of the myeloid/lymphoid balance towards myeloid population is being observed following injuries, such a infection, inflammation, and irradiation [65]. This balance is controlled by homeostasis of each subpopulation.

**Fig. 5:**
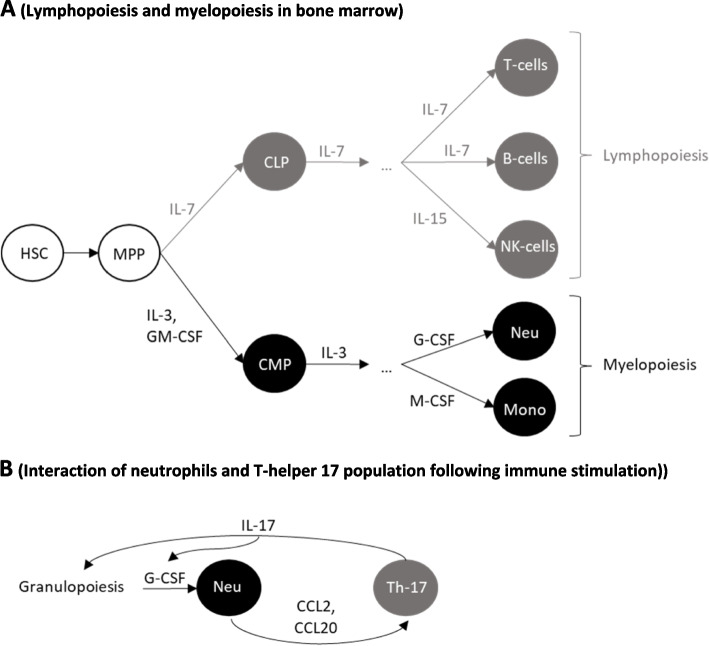
**A** Lymphopoiesis and myelopoiesis pathways from common progenitors. Legend: HSC, MPP, CLP, CMP, Neu, and Mono: hematopoietic stem cells, multipotent progenitors, common lymphoid progenitors, common myeloid progenitors, neutrophils, and monocytes, respectively. From CLP and CMP, lymphoid and myeloid differentiation and maturation are separately processed. **B** Interactions of neutrophils and Th-17: T-helper 17 cells. Legend: Chemokines secreted by neutrophils induce IL-17 secretion by Th-17. IL-17 induces granulopoiesis, upregulates G-CSF, thereby inducing the differentiation and mobilization of neutrophils from progenitors within the bone marrow. This results in increasing neutrophil levels in the blood

In hematopoiesis, multipotent progenitors (MPP) differentiate either into common lymphoid progenitors (CLP) or common myeloid progenitors (CMP), which are the progenitors of mature lymphoid and myeloid cells, respectively. The probability that a MPP follows a CLP rather than CMP route is regulated by mature lymphoid and myeloid cells in the periphery. After this step, lymphoid and myeloid populations develop and maturate in distinct pathways, which are regulated by different growth factors (cytokines) (Fig. 5A) [65].

Interactions between lymphoid and myeloid populations take place following immune stimulation. Recent evidence in humans demonstrated cross-communications between activated neutrophils and T-helper 17 CD4 + T-cells. Chemokine secretion by stimulated neutrophils recruits T-helper 17 lymphocytes to injury sites, stimulating IL-17 secretion. IL-17 upregulates G-CSF and enhances neutrophil production in the bone marrow, in addition to their mobilization to the periphery (Fig. 5B). During inflammation, interactions between myeloid and lymphoid populations contribute to relations between innate and adaptive immune responses. Yet, it is still unclear whether such interactions also directly happen following RT.

Several lymphoid/myeloid ratios have been associated with response to radiotherapy, while being used in the clinical context to predict treatment efficacy, such as neutrophil to lymphocyte ratio (NLR) and lymphocyte to monocyte ratio (LMR). NLR is the ratio of neutrophil over lymphocyte levels in the peripheral blood. NLR has been used as an inflammation marker. A high NLR was shown associated with poor prognosis in several cancers, such as esophagus cancers. NLR has also been used in estimating dose exposure following radiation accidents. LMR is the ratio of monocyte over lymphocyte levels in the peripheral blood. LMR reflects the degree of systemic inflammation, which has been associated with long-term prognosis in cancer patients [66]. A high LMR of 4.25 was reported to be associated with favorable disease-free survival in locally advanced breast cancer patients [67].

### Quantitative modeling of leucocyte kinetics following radiation

Preliminary data and modeling approaches of radiation-induced leucopenia suggest that recovery kinetics and mechanisms differ among white cell lineages. Iterative hypothesis-generating processes can further help provide insights on the ill-defined mechanisms of radiation-induced leucopenia. Modeling approaches were separated into single time-point analysis and time-series with kinetics based on acquisitions at multiple timepoints. Because modeling accuracy and generalizability are dependent on the initial assumption and may thus suffer from overfitting, integration of prior knowledge and model validation are required in order to minimize overfitting, thereby improving model accuracy.

#### Single time-point analysis

Radiation causes perturbations of leucocyte subpopulations. These perturbations can be influenced by radiation parameters (dose, fractionation, rate, and volume of irradiated tissue), individual characteristics (body weight, age, gender, and cardiac output), or disease status (tumor location). Modeling selects essential parameters and quantifies their effect on leucocyte subpopulations.

Multiple studies on radiation-induced leucopenia have focused on radiation-induced lymphopenia using single time-point analysis (in addition to baseline). The endpoints were either early lymphocyte counts during or shortly after irradiation (acute effect), late lymphocyte counts (chronic effect), or lymphopenia severity (Grade 1–4). By way of illustration, in one study [68], modeling was simply based on piecewise linear and exponential models so as to analyze the relationships between lymphocyte nadir and dose in the head and neck area [68]. In another study [69], a hybrid deep learning model was employed to screen several parameters for their impact on lymphocyte counts following radiotherapy in esophagus cancer patients. In addition to these statistical models, blood flow models have been considered under the assumption that radiation-induced lymphopenia was caused by exposure of blood circulating lymphocytes [46, 70].

Models built on patient data (with tumors) are intrinsically limited in that they cannot determine whether radiation-induced lymphopenia is due to systemic effects (or the presence of other concomitant treatments such as chemotherapies) or an interaction between tumor and radiation beams. Models built on healthy individuals can explore the systemic effect of irradiation on lymphocytes and leucocyte subpopulations. Animal models have used total body irradiation in healthy total Rhesus macaques [43, 44], or focal brain irradiation in mice [71]. However, these models relied on single time-point analyses.

#### Time-series analysis

Radiation-induced leucopenia results from multiple mechanisms including changes in circulating cells, cell production from primary organs, cell maturation in secondary organs, cell distribution in the body, and cellular interactions, as well. Leucocyte recovery from the acute radiation effects on circulating cells is being observed after a certain time that depends on homeostatic regulation.

The physiologic homeostasis of leucocytes is regulated independently among leucocyte subpopulations. Similarly, our modeling approach was based on the assumption that myeloid and lymphoid, B and T-, as well as naïve and memory T-cell population kinetics are regulated separately following radiotherapy. To estimate the balance of myeloid/lymphoid kinetics, NLR and LMR may also be applied as parameters. Based on data from healthy macaques and mice, a second assumption was that initial parameters influence acute radiation-induced leucopenia rather than the recovery phase. An alternative assumption may consider the impact of initial parameters on recovery in the event the first model lacked consistency with observational data.

A study in rhesus macaques showed leucocyte subpopulations in both lymphoid and myeloid lineages recovered following acute depletion induced by a single lethal dose of total body irradiation in surviving macaques [44]. Cells count post irradiation were not significantly different from those of the non-irradiated group and remained stable for more than a year. This suggest that post-radiation homeostasis could be assimilated to the normal physiological homeostasis. However, recovery is delayed and is dependent on radiation conditions (dose, rate, volume). In some cases, post-irradiation homeostasis only partially recovers, i.e., to a lower level than before irradiation. Modeling can help to identify the steady post-irradiation level and to screen for the radiation conditions that participate in this perturbation.

##### Phenomenological modeling approach

**Fig. 6:**
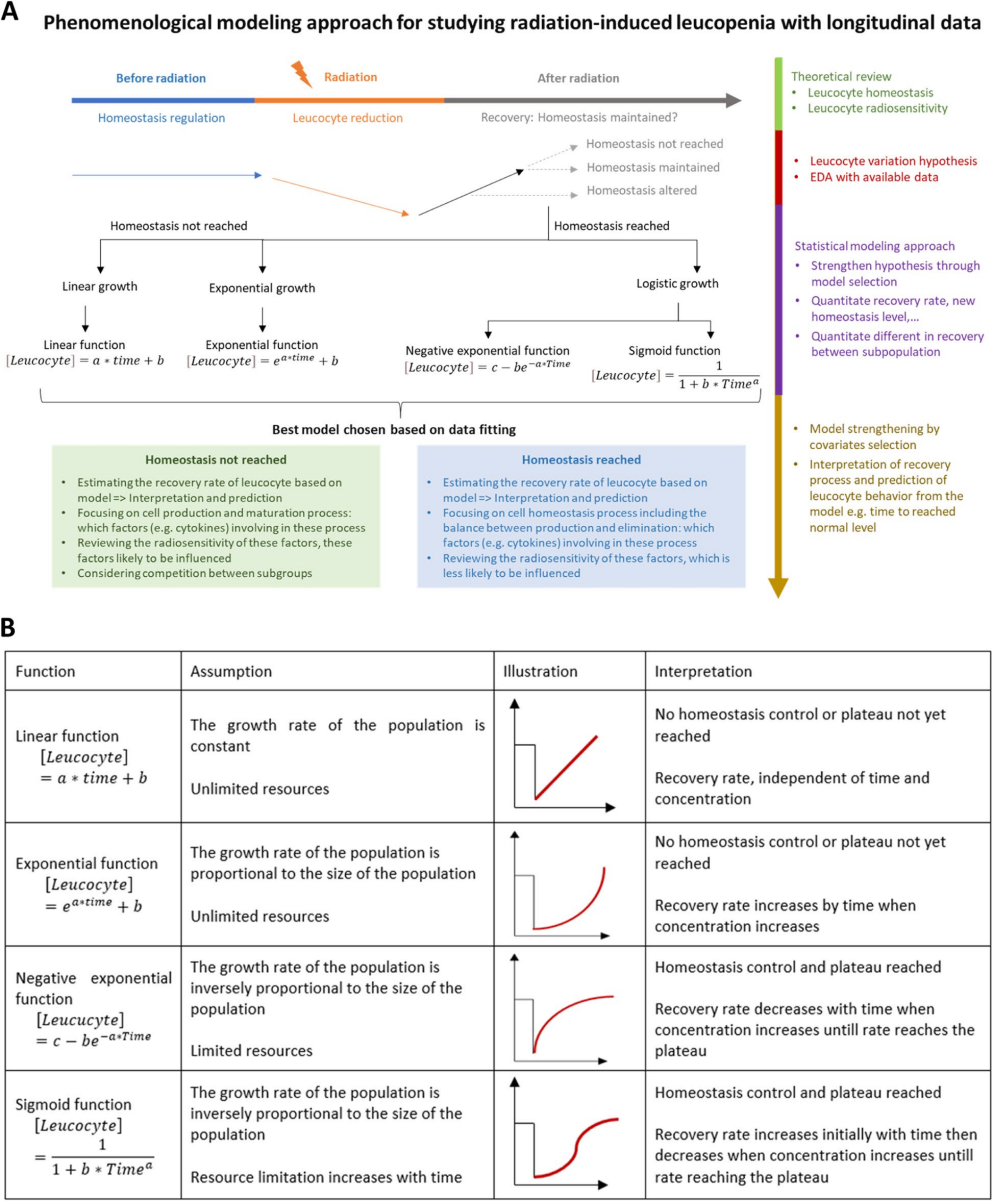
**A** Phenomenological modeling approach of radiation-induced leucopenia based on longitudinal data. **B** Functions for possible phenomenological modeling approaches. Legend: Depending on the data trend (growth trend), appropriate function types can be used for data fitting. In agreement with the biological homeostasis theory and radiosensitivity, the function type would provide information regarding the characteristics of the recovery processes

Phenomenological modeling is based on information extracted from data without trying to clarify the underlying mechanisms that led to the observed data [72]. It informs on underlying data-generating mechanisms and data interpretation. Phenomenological modeling from radiation-induced leucopenia data could help better understand its mechanisms. In regression modeling, a specific function is declared and fitted with the available data in the way that minimizes the distance between the data and predicted data distributed according to a regression function. Based on the recovery pattern, several linear and nonlinear models may be considered using functions (Fig. 6B). Although this approach arises from experimental data rather than prior biological theory, some links between suggested functions and homeostasis processes are likely identified for model interpretation.

Choosing the best model could be based on the following two approaches: 1) exploratory data analysis in which data are summarized with their main characteristics by data visualization; 2) model selection criteria including mean squared error, Akaike information criterion, or Bayesian information criterion. The best model provides information about the following: 1) recovery pattern; 2) whether a stable point (plateau) was reached during the observation period; 3) whether the stable point reached was the same as before treatment, which implies that homeostasis is being either achieved or altered; 4) if a stable point was not reached, it could mean that either homeostasis control and homeostasis were dysregulated, or that the observation period was not long enough for homeostasis regulation. The framework for phenomenological modeling is generalized in Fig. 6A.

##### Mechanistic modeling approach

Mechanistic modeling is based on understanding the mechanism underlying the data. This approach is useful for hypothesis verification or for subsequently refining mechanistic models [72]. The most common mechanistic modeling type consists of compartment models that describe the variation of quantities (here leucocyte counts) by ordinary differential equations (ODE). This is a well-known approach in studying dynamics and system evolution with time. Compartments, illustrated as boxes, and transfer rates showing changes among compartments for each time unit, are given by differential equations. The general form of each differential equation is the following:

$$\frac{d(variable)}{dt}=input-output$$

**Fig. 7:**
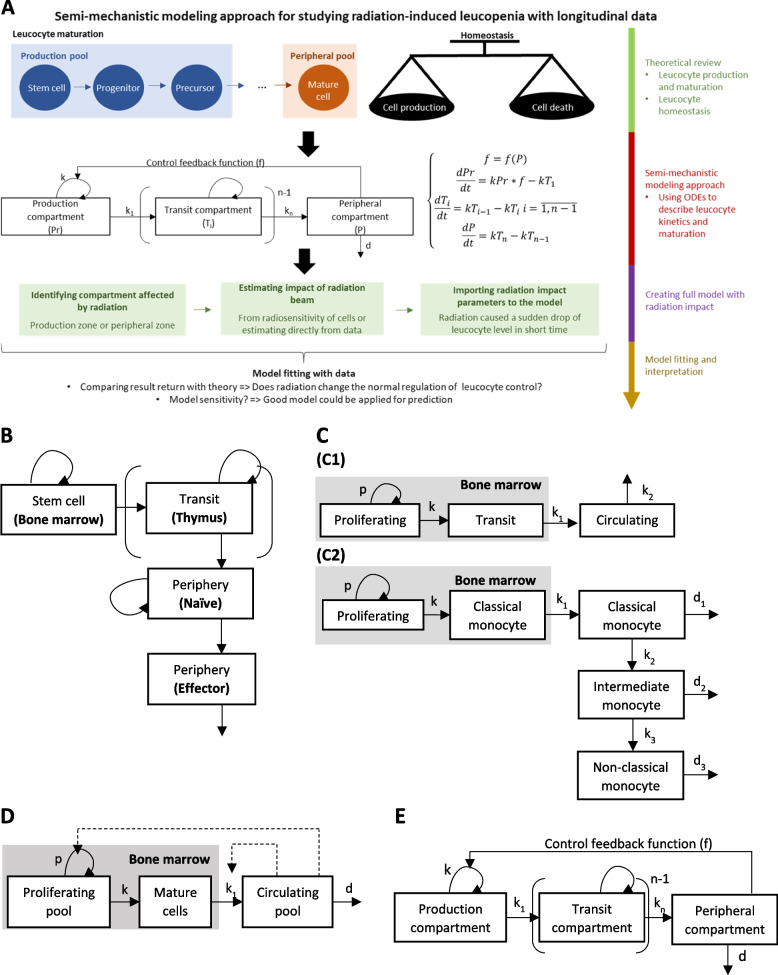
**A** A semi-mechanistic modeling approach framework for investigating radiation-induced leucopenia with longitudinal data. Legend: A model structure (graph and ODE) for cell kinetics was built based on theory of cell production, maturation, and homeostasis. Based on the cell kinetics model, target compartments and parameters of radiation beams must be defined (based on theory and treatment planning system). **B** Structural model of lymphocyte population kinetics. Legend: This model includes: 1) a stem cell compartment with proliferation (here the bone marrow) and output, yet no input; 2) the thymus in cases of T-cells, with input, proliferation, and output of cells; 3) the peripheral pool of immunocompetent mature T and B lymphocytes, which are divided in transit compartments; 4) an effector compartment formed by antibody-producing plasma cells, which constitutes a simple transit compartment, with input and output (cell loss), yet no proliferation. **C** Structural model of myeloid population kinetics: (C1) structural model of neutrophil population kinetics. The model considers mitotic neutrophil precursors as a single proliferating pool. Cells in this pool proliferate at a mean rate p. After the last mitosis, the cells enter the transit pool at a rate k. Transit neutrophils remain for a period in the bone marrow before being released into the circulating pool at rate k_1_. The cells either leave this pool in the direction of other marginal organs or die by apoptosis at rate k_2_. (C2) Structural model of monocyte population kinetics. The model depicts monocytes in the bone marrow, where their precursors proliferate at rate p and mature at rate k. Mature monocytes are released from the bone marrow at rate k_1_ into the circulation. In blood, monocytes either mature into intermediate monocytes at rate k_2_ or disappear from the blood (by death or by moving to other organs) at rate d_1_. Intermediate monocytes either differentiate into long-lived non-classical monocytes at rate k_3_ or disappear from the blood at rate d_2_. Non-classical monocytes are the final differentiation stage, which disappear from the blood at rate d_3_. **D** Simplified structural model of myeloid cell population kinetics following cell depletion. This model comprises a proliferating pool, mature pool in bone marrow, and circulating pool. A negative feedback loop was added, in which the proliferating rate and transfer rate from bone marrow to blood were negatively controlled by the circulating pool size. **E** Compartment description of Friberg’s model. The model consists of a proliferating compartment that is sensitive to drugs, in addition to three transit compartments that represent maturation, and a compartment of circulating blood cells

In 1986, a preliminary model described lymphocyte kinetics in rodents with either three compartments (B-cells) or four compartments (T-cells) (Fig. 7B) [73]. Based on this model, data from experiments of post-irradiation lymphocyte kinetics were employed for model fitting in order to investigate whether physiologic homeostasis was being observed following irradiation. This first step can be instrumental enabling us to establish more modern and more complex mechanistic models that integrate the multiple steps of maturation and feedback control on the proliferation [74].

Myeloid cell kinetics is less complex than that pertaining to lymphocytes, given that there is no recirculation. Several structures of mechanistic models have been proposed, such as neutrophil or monocyte kinetic models developed based on myelopoiesis, as well as their dynamics in the blood (Fig. 7C). Parameter estimation based on human data using deuterium-labeled glucose was revealed to be well-fitted with theoretical data [58, 75].

Comparing model fitting results of healthy or leucopenic individuals demonstrated that the proliferating rate and transfer rate from the bone marrow to blood increase when the circulating pool is reduced. This change leads to the recovery of the circulating pool, in agreement with the homeostasis theory. Based on this idea, a new model structure is proposed with some modifications from the known mechanistic model. A negative feedback loop was added, in which the proliferating rate and transfer rate were negatively controlled by the circulating pool size (Fig. 7D). The negative feedback loop represents homeostatic regulation: in leucopenic conditions, when the circulating pool size is smaller than the physiological level, the turnover rate of the proliferating pool increases accordingly to help the circulating pool recover [76]. On the other hand, when the circulating pool exceeds the physiological level, the negative feedback loop drives the proliferating pool to lower its turnover rate until the circulating pool returns to physiological level [76].

A feedback loop has already been applied to models of neutropenia induced by chemotherapy agents since 2002 (Friberg’s model, [77]). Because mechanisms of neutropenia induced by chemotherapy or radiotherapy both affect the bone marrow [15, 77], the Friberg’s model may be applied to radiation-induced neutropenia, and a structural model may thus be built (see compartmental graph in Fig. 7E) [77].

Feedback is illustrated as an inversely proportional function of the circulating pool size to the proliferating pool turnover rate [77–79]. The feedback loop in Friberg’s model [77] was described as:

$$f\left(C\right)={\left(\frac{{C}_{0}}{C}\right)}^{\gamma }; k={k}_{0}{\left(\frac{{C}_{0}}{C}\right)}^{\gamma }$$

where f(C) is the negative feedback function; k is the turnover rate of proliferating pool; k_0_ is the turnover rate of proliferating pool at steady state; C is the circulating pool size; C_0_ is the circulating size at steady state; and γ is the coefficient of homeostasis control. Following this function, turnover rate k would increase when C is lower than C_0_ and return back to k_0_ when C is equal to C_0_.

##### Example: radiation-induced neutropenia in non-human primates following total body irradiation

**Fig. 8:**
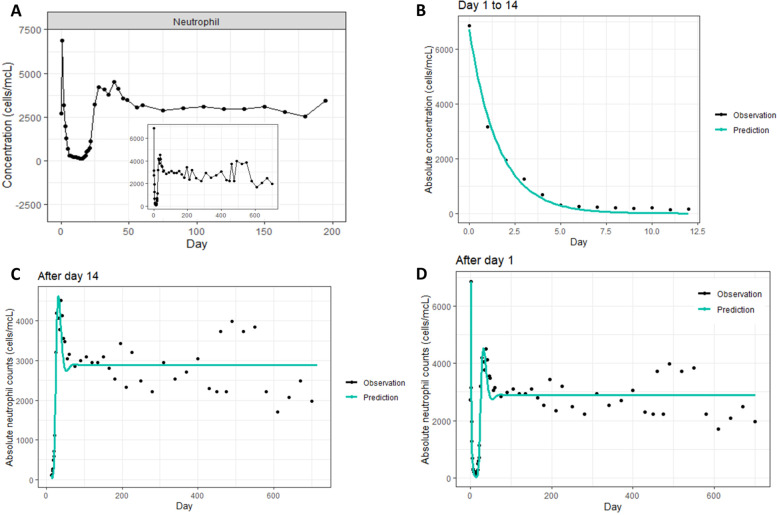
Radiation-induced neutropenia following total body irradiation in rhesus macaques. **A** Data extracted from [43]; **B** Exponential model fitting for the early phase; **C** Friberg’s model fitting for the late phase; **D** Model fitting for the whole time-series data. The black dots and blue line in (B), **C**, and **D** represent data and prediction, respectively. Data illustration and modeling were processed based on extracted data from Farese et al. 2015 [43]

Both phenomenological and mechanistic models likely contribute to understanding radiation-induced leucopenia. To illustrate this point, we have provided an example using data extracted from a study of radiation impact on circulating neutrophils in macaques [43]. From the data extracted, neutrophil kinetics following irradiation were separated into two periods: [1] An early phase (Day 0–14) showing a rise at Day 1 with exponential decline from Day 1 to Day 14; [2] a late phase (from Day 14) showing sigmoid-like increase with a slight rebound before reaching a steady level (Fig. 8A).

For the early phase, a phenomenological modeling approach was applied using the exponential model:

$${C}_{t}={C}_{0}{e}^{-kt}$$

where ${C}_{t}$ is the neutrophil concentration at Day t + 1; ${C}_{0}$ is the neutrophil concentration at Day 1; t is the day after Day 1; k is the parameter estimated relating to the neutrophil reduction rate. For the late phase, the semi-mechanistic modeling approach was applied using the Friberg’s model (Fig. 7E), assuming that the neutrophil concentration in the circulating pool induced negative feedback on the proliferation rate in the proliferating pool (detailed parameters estimation is illustrated in supplementary Table 2). For both phases, the model returned good parameter estimation and well-fitted prediction curves (Fig. 8B-Fig. 8C). The phenomenological exponential model for the early phase might be interpreted as a semi-mechanistic model where during the first 15 days, there is no neutrophil input to blood from Day 1–14, with the clearance (death rate) driving neutrophil kinetics.

From the two-phase models, a whole time-series model could be inferred where: [1] radiation caused a reduction of k to 0 from Day 1; [2] the k value increased gradually from Day 1 to Day 14 so as to reach a stable level. The model structure is illustrated in Supplementary Fig. 5. The model for the whole time-series returned prediction curves that were well-fitted with the data (Fig. 8D).

Finally, the model structure and parameter are consistent with prior knowledge. Indeed, in humans, neutrophil concentration in blood is dependent on cytokines (G-CSF, IL-17, IL-23…), which regulate the production and maturation of neutrophils in the bone marrow. Modeling results showed that radiation-induced neutropenia was likely caused by a proliferation lack rather than by a direct impact on the circulating pool. As suggested, full recovery to nearly initial levels could be reached after about 14 days.

##### Variable screening

Radiation parameters (dose, fractionation, rate, volumes, etc.) and individual status (cardiac output, body weight, age, gender, etc.) might interfere with homeostatic regulation of leucocyte subpopulations, thereby resulting in chronic radiation-induced leucopenia. Chronic leucopenia may be detected by stratifying data on parameter values, while relevant parameters could be imputed into the model.

By way of illustration, assuming that cell population kinetics after irradiation follows a linear model C = C0 + kt, where C is the cell concentration at certain time t, and C0 the concentration during irradiation. The parameter k of the linear function is estimated with: k = k1 when radiation dose d = d1, k = k2 when radiation dose d = d2, and k = k3 when radiation dose d = d3. The radiation dose effect on cell recovery can be assessed using a new function f(d) = k in order to represent the correlation between k and d. Model comparison using AIC can then be applied to select the best f(d), as well as compare the new (with variable imputation) and initial models (without variable imputation) with respect to impact of radiation dose.

##### Individual based modeling

**Fig. 9:**
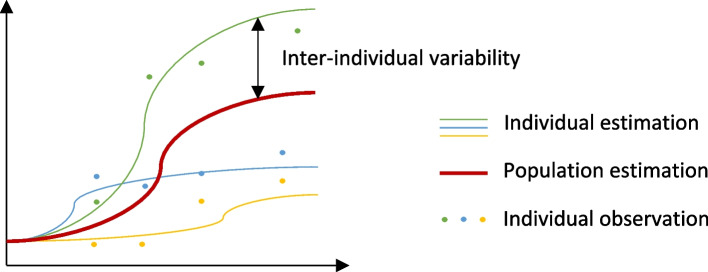
Illustration of how individual-based modeling is applied in modeling. Legend: In conventional modeling, a common model is evaluated as the mean of the whole population. In individual-based modeling, population estimates are based on the estimation of each individual

Conventional modeling approaches predict the mean behavior of a whole population. When dealing with longitudinal replication data, this approach neglects the interaction between factors for each individual. Individual-based modeling is a hierarchical modeling approach that accounts for a high complexity degree among individuals, as well as interactions among individuals, where populations are analyzed as a composition of discrete individual organisms in which each individual has a set of state variables, such as longitudinal time points (Fig. 9) [80]. Studies of leucocyte subpopulation recovery following radiation in rhesus macaques revealed a high variability among individuals, suggesting effect of radiation-induced leucopenia to be heterogeneous [44]. Individual-based modeling appears particularly useful with heterogenous data, where a conventional approach would deal with confounding factors, and thus render the population trends misleading with respect to what truly happens within the population at the individual level. Although powerful, individual-based modeling is not always possible, given that it requires access to individual data, which is not always possible when retrieving literature data. In addition, more complicated algorithms and machines are needed for individual-based modeling application. (Fig. 9)

### Model validation

The use of modeling in biology often raises questions of how well the model likely applies to the real-world setting. The optimal way is to perform model validation using large sets of independent data. Model validation for small datasets would require either goodness-of fit, cross validation, or both [81]. Goodness-of-fit estimates the randomness of the distribution of residuals, which represent the portion of the validation data that cannot be explained by the model [81]. Cross validation partitions the data into complementary subsets, performing analyses on one subset, and validation on another subset, iteratively. Cross-validation can detect overfitting, thus providing insights on how the model would generalize to an independent dataset [81]. In addition to statistical validation, prediction obtained from bio-mathematical models must always be challenged for consistency with prior knowledge.

## Conclusion

Modeling of radiation-induced leucopenia, in combination with prior knowledge of leucocyte homeostasis and radiosensitivity, opens new approaches to understand the mechanisms behind leucocyte variation following radiation. With availability of new preclinical and clinical data of leucocyte kinetics following radiation, modeling likely provides more insights in underlying mechanisms. Modeling can not only assess the impact of radiation parameters on leucocyte subpopulations, but also their recovery following acute leucopenia, which can be performed by combining prior physiology knowledge and available data. Understanding leucocyte physiology is critical for both model development and interpretation. Pioneering studies were mainly focused on radiation effects on a single time point, yet without considering the recovery of leucocyte counts with time. This review contributes to defining the impact of radiation parameters on both a single-time point of acute effects and subsequent recovery of leucocyte subpopulations. It may facilitate the optimization of cancer treatments by predicting leucocyte levels depending on radiation modality, i.e., X-rays or hadrontherapy, and of the combination of radiotherapy and immunotherapy. In particular, assessing leucocyte count variations with time following radiation can also help define the optimal time for immunotherapy initiation. Although promising, modeling must be applied carefully with proper data calibration. To date, modeling of leucocyte kinetics following irradiation only considers changes in leucocyte counts, without taking account of their function. This is a first step towards modeling and understanding the effects of fractionated radiotherapy, whereas more data are required in view of a more reliable prediction of cancer treatment outcomes.

## Supplementary Information

**Additional file 1.**

## Data Availability

All data analyzed during this study were included in this published article.

## Abbreviations

TCR: T-cell receptor
BCR: B-cell receptor
LNs: Lymph nodes
SLOs: Secondary lymphatic organs
IL: Interleukin
MHC: Major histocompatibility complex
G-CSF: Granulocyte colony-stimulating factor
M-CSF: Macrophage colony-stimulating factor
GM-CSF: Granulocyte–macrophage colony-stimulating factor
SDF1: Stromal derived factor-1
CXCR4: CXC-receptor 4
CCR2: C–C chemokine receptor 2
NLR: Neutrophil to lymphocyte ratio
LMR: Lymphocyte to monocyte ratio
MPP: Multipotent progenitors
CLP: Common lymphoid progenitors
CMP: Common myeloid progenitors
ODE: Ordinary differential equations
