# Supplementary material for: Beyond lymphopenia, unraveling radiation-induced leucocyte subpopulation kinetics and mechanisms through modeling approaches
Source: J Exp Clin Cancer Res. 2023 Feb 22;42:50. doi: 10.1186/s13046-023-02621-4 (PMC9945629; doi:10.1186/s13046-023-02621-4)
Supplement: Supplementary file 1 — Additional file 1. [file 13046_2023_2621_MOESM1_ESM.pdf]

Supplementary 1: Lymphocyte recirculation model in rodents

Ganusov VV, Auerbach J. Mathematical Modeling Reveals Kinetics of Lymphocyte Recirculation in the Whole Organism. *PLoS Comput Biol* 2014; **10**: e1003586.

Thoracic duct lymphocyte recirculation mathematical model was developed in rodents (Ganusov *et al.*), by assuming blood as a major compartment that delivers lymphocytes to other tissues, then lymphocytes exit those tissues to the blood. The model structure and parameters are presented in the figure below.

A

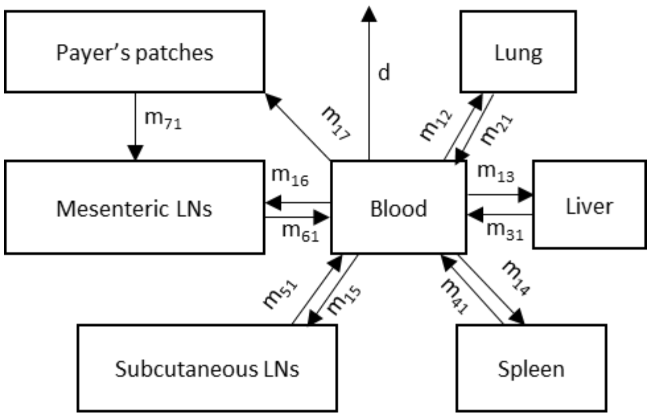

B

Parameter	Value (min^{-1})	Parameter	Value (min^{-1})
m_{12}	1·83	m_{21}	2·17
m_{13}	0·41	m_{31}	1·14
m_{14}	0·056	m_{41}	0·007
m_{15}	0·026	m_{51}	0·0034
m_{16}	0·0106	m_{61}	0·0034
m_{17}	0·0053	m_{71}	0·0034

Supplementary figure 1. (A) Structural description by boxes of lymphocyte recirculation model in rodents with i compartments denoted as: 1) Blood; 2) Lung; 3) Liver; 4) Spleen; 5) Subcutaneous LNs; 6) Mesenteric LNs; and 7) Payer's patches. Intercompartmental transfer denoted as: m_{1i} is transfer rate from blood to tissue i; m_{i1} is transfer rate from tissue i to blood, except for m_{71} is transfer rate from tissue 7 to tissue 6. (B) Parameter estimation retrieved from Ganusov *et al.*

The change in each compartment in each time unit is given by a differential equation. The general form of each differential equation is of the form:

$$\frac{d[Blood]}{dt} = \sum_{i=1}^{6}(m_{i1}[Tissue\ i] - m_{1i}[Blood]) - m_{17}[Blood]$$

$$\frac{d[Tissue\ i]}{dt} = m_{1i}[Blood] - m_{i1}[Tissue\ i]\ for\ i = \overline{2,5}$$

$$\frac{d[Tissue\ 6]}{dt} = m_{16}[Blood] + m_{71}[Tissue\ 7] - m_{61}[Tissue\ 6]$$

$$\frac{d[Tissue\ 7]}{dt} = m_{17}[Blood] - m_{71}[Tissue\ 7]$$

[x] is the lymphocyte concentration in compartment x; compartment i denoted as: 1) Blood; 2) Lung; 3) Liver; 4) Spleen; 5) Subcutaneous LNs; 6) Mesenteric LNs; and 7) Payer's patches

The first simulation scenario is to check the distribution of lymphocyte in non-pathogenic individual. In this scenario, a certain amount of lymphocyte was injected into circulation blood. Without any immune stimulations, lymphocyte would distribute in tissue and reach the steady state by time. Assuming there is no cell death during the distribution time, the result gotten from simulation was depicted in Supplementary figure 2.

At the stable state, the proportion of lymphocytes in the rodents' body obtained from simulation is illustrated in the table below. Only 4.15% of lymphocytes in the body were in the blood at any time in normal conditions. A large number of lymphocytes were stored in the spleen and lymph nodes, indicating that when

there is a sudden depletion of lymphocytes in circulating blood, lymphocytes from those organs could recirculate to maintain blood levels.

The second simulation scenario is to depict the kinetics of lymphocytes following an acute reduction in blood compartment. We simulated the situation after 20 min at the stable state, a sudden reduction of lymphocytes in the blood compartment of different levels ranging from 8% to 80% compared to baseline appeared. In case all other organs function normally, blood lymphocyte levels reach more than 80% from baseline after 3 hours even depleting almost all lymphocytes in the blood compartment (Supplementary figure 3).

Supplementary table 1. Lymphocyte distribution in the blood and tissues following first scenario simulation

Organ	Blood	Lung	Liver	Spleen	SCLNs	MLN	PPs
% of lymphocyte	4.15	3.50	1.49	33.21	31.74	19.41	6.60

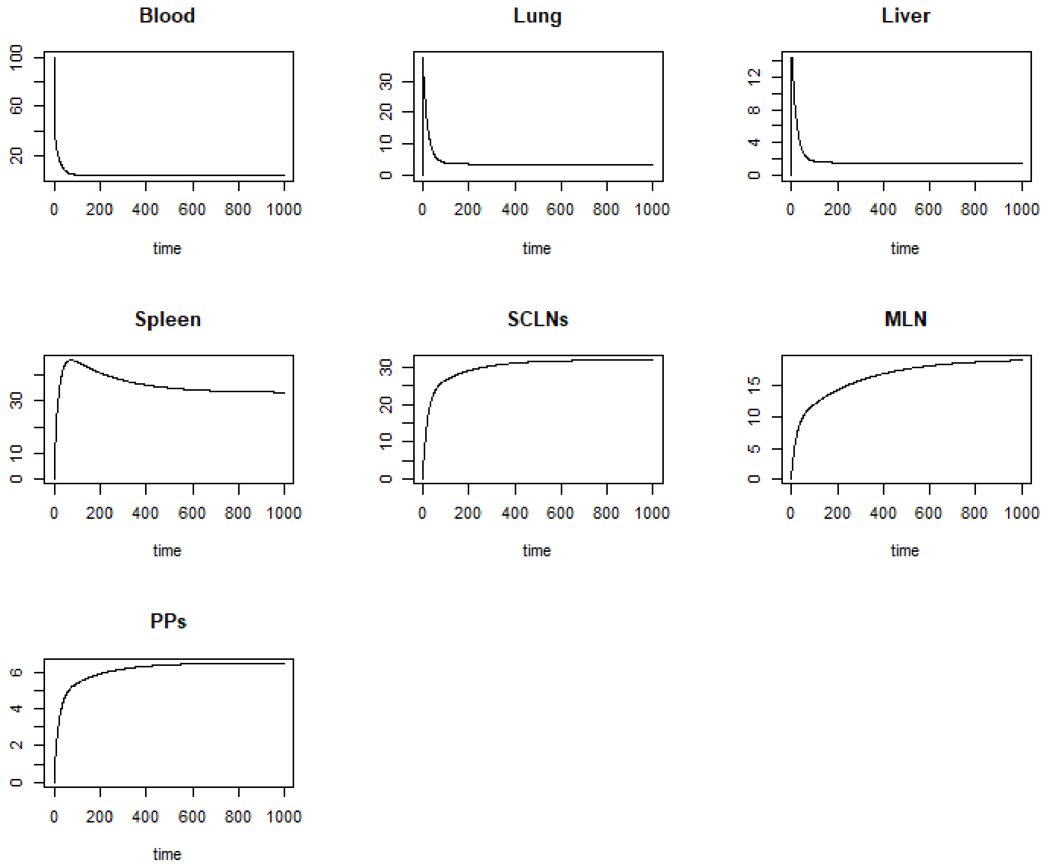

Supplementary figure 2. Simulation result of the first scenario.

Legend: The x-axis represents time in minutes, and the y-axis the proportion of lymphocytes in each organ compared with all lymphocytes of the body

A (Drop of lymphocytes in the blood compartment to 80% of their initial value)

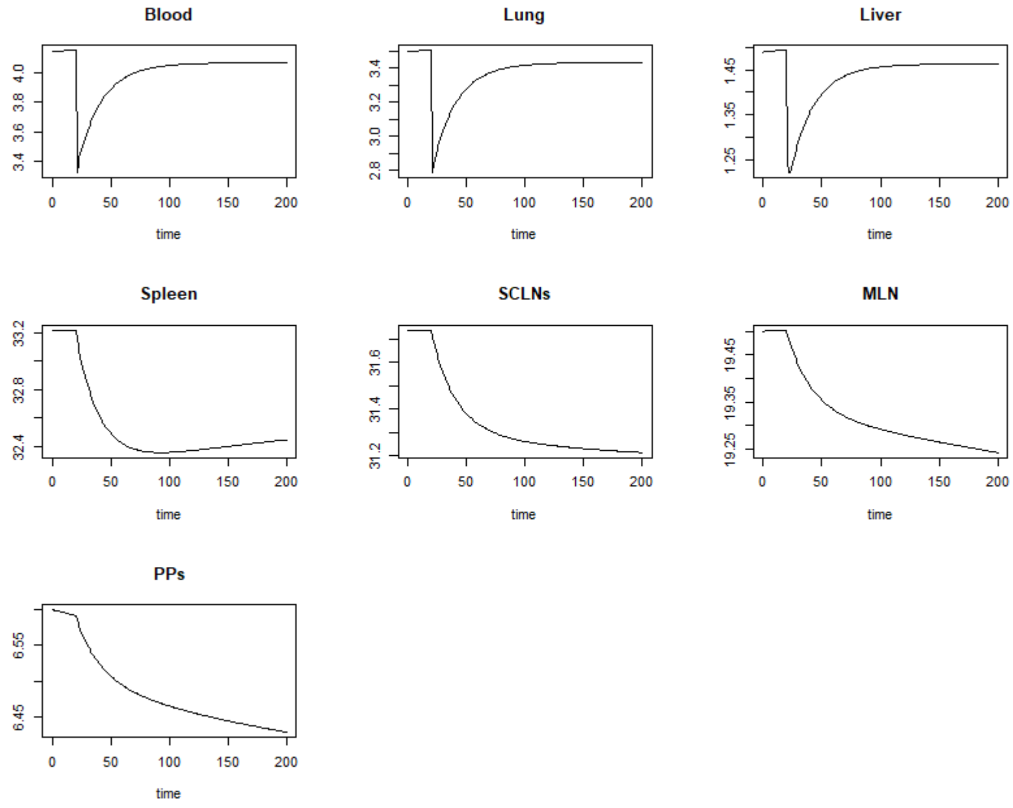

B (Drop of lymphocytes in the blood compartment to 56% of their initial value)

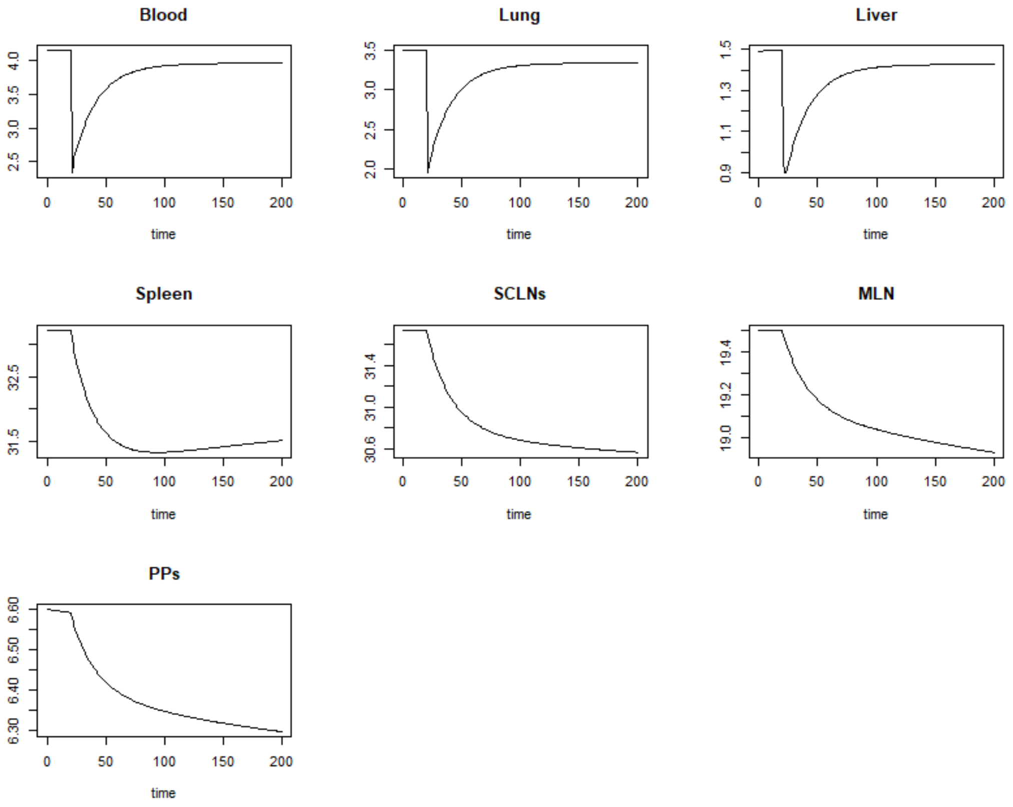

C (Drop of lymphocytes in the blood compartment to 32% of their initial value)

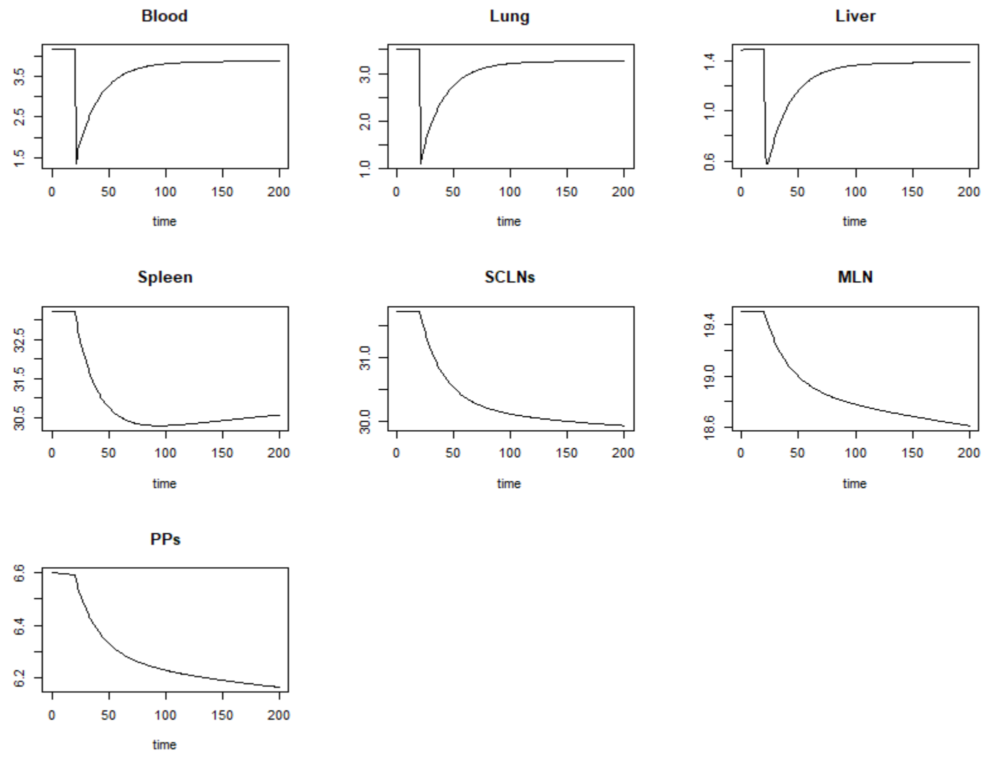

D (Drop of lymphocytes in the blood compartment to 8% of their initial value)

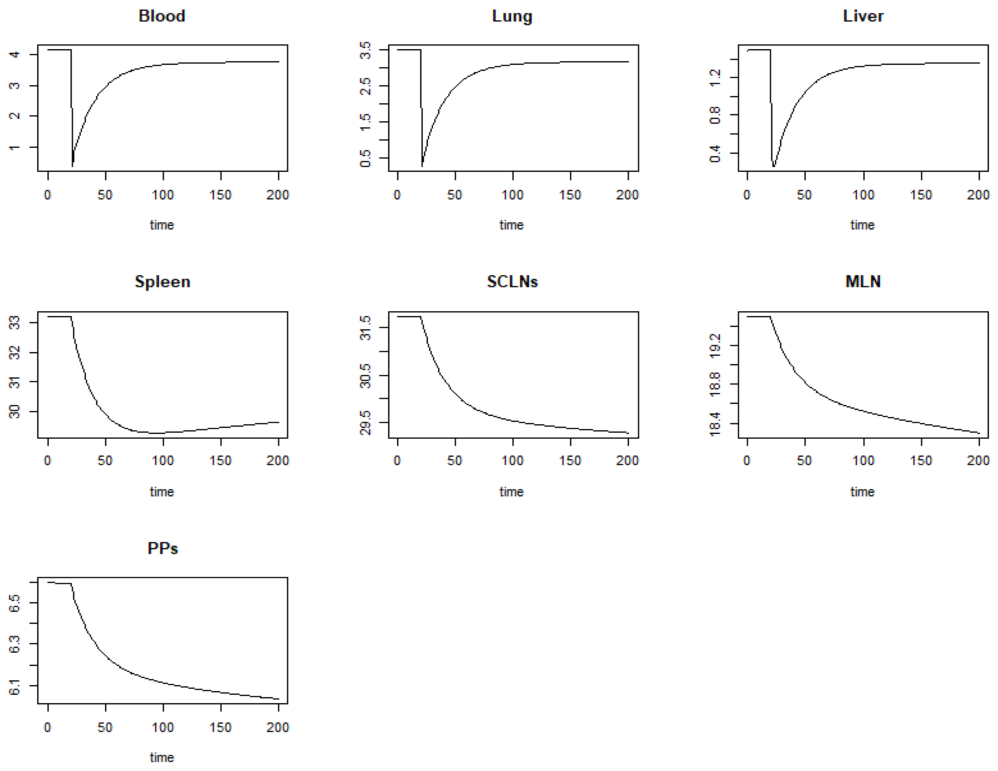

Supplementary figure 3. Simulation of a scenario in which there is a sudden drop of lymphocytes in the blood compartment.

Legend: (A) Drop to 80%; (B) Drop to 56%; (C) Drop to 32%; (D) and Drop to 8%. The x-axis represents time in minutes, and the y-axis the proportion of lymphocyte in each organ compared with the initial value before acute reduction in the blood.

Supplementary 2: Radiation-induced neutropenia in Rhesus macaques following total body irradiation

Supplementary table 2. Parameter estimation for early stage (exponential model)

C_0		k	
**Value**	**SE (%RSE)**	**Value**	**SE (%RSE)**
6713.1	194.6 (2.90%)	0.63	0.03 (5.45%)

Supplementary table 3. Parameter estimation for late stage (Friberg's model)

**Cell population**	**Baseline (cells/μL)**	$\boldsymbol{k = \mathrm{d}}$		$\boldsymbol{\gamma}$		$\boldsymbol{C_{ss}}$		**s2 over baseline**
		**Value**	**SE (%RSE)**	**Value**	**SE (%RSE)**	**Value**	**SE (%RSE)**	
Neutrophil	2715.44	0.71	0.03 (4.49%)	0.15	0.01 (3.63%)	2891.16	66.98 (2.32%)	106.47%

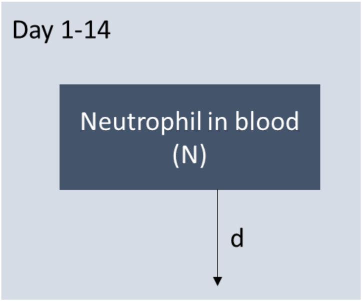

$$\frac{dN}{dt} = d\ (death\ rate)$$

$$N_0 = 6713.1 cells/\mu L d = 0.63\ (cells/\mu L/day)$$

Supplementary figure 4. Semi-mechanistic model structure for early stage of radiation-induced neutropenia following TBI in Rhesus macaques

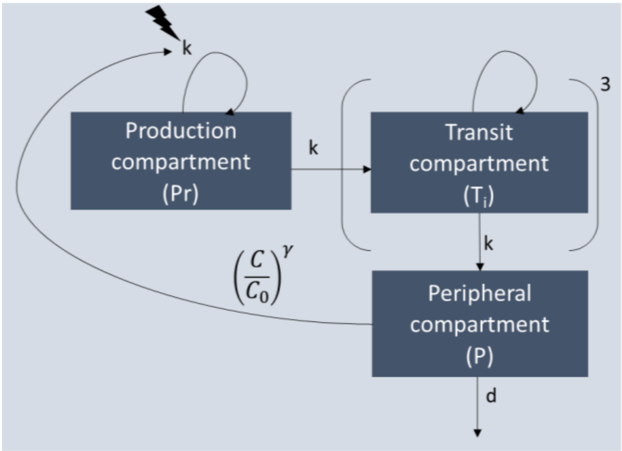

$$\frac{dk}{dt} = a * k * \left(1 - \frac{k}{k_{max}}\right);$$

$$k_0 = 0.1\ ; k_{max} = 0.71 (cells/\mu L/day)$$

$$a = (k_0 * Day_{Nadir})^{-1} = 0.67 (cells/\mu L)^{-1}$$

Supplementary figure 5. Semi-mechanistic model structure for whole time-series of radiation-induced neutropenia following TBI in Rhesus macaques
